# Anatomical relationships between serotonin 5-HT_2A_ and dopamine D_2_ receptors in living human brain

**DOI:** 10.1371/journal.pone.0189318

**Published:** 2017-12-08

**Authors:** Tatsuya Ishii, Yasuyuki Kimura, Masanori Ichise, Keisuke Takahata, Soichiro Kitamura, Sho Moriguchi, Manabu Kubota, Ming-Rong Zhang, Makiko Yamada, Makoto Higuchi, Yoshinori Okubo, Tetsuya Suhara

**Affiliations:** 1 Department of Functional Brain Imaging Research, National Institute of Radiological Sciences, National Institutes for Quantum and Radiological Science and Technology, Chiba, Japan; 2 Department of Neuropsychiatry, Nippon Medical School, Tokyo, Japan; 3 Department of Clinical and Experimental Neuroimaging, Center for Development of Advanced Medicine for Dementia, National Center for Geriatrics and Gerontology, Obu, Japan; Banner Alzheimer’s Institute, UNITED STATES

## Abstract

Serotonin 2A (5-HT_2A_) receptors and dopamine D_2_ receptors are intimately related to the physiology and pathophysiology of neuropsychiatric disorders. A large number of studies have reported the effectiveness of psychotropic agents targeting 5-HT_2A_ and D_2_ receptors in these disorders. In addition to the individual functions of these receptors, the interaction between the two neurotransmitter systems has been studied in the living brain. However, little is known about their regional relationship in individual human brains. We investigated regional relationships between 5-HT_2A_ and D_2_ receptors using positron emission tomography (PET) and a bicluster analysis of the correlation matrix of individual variation in the two receptor densities to identify groups of distinctive regional correlations between the two receptors.

**Methods:**

Seven healthy volunteers underwent PET scans with [^18^F]altanserin and [^11^C]FLB 457 for 5-HT_2A_ and D_2_ receptors, respectively. As a measure of receptor density, a binding potential (*BP*) was calculated from PET data for 76 cerebral cortical regions. A correlation matrix was calculated between the binding potentials of [^18^F]altanserin and [^11^C]FLB 457 for those regions. The regional relationships were investigated using a bicluster analysis of the correlation matrix with an iterative signature algorithm.

**Results:**

We identified two clusters of regions. The first cluster identified a distinct profile of correlation coefficients between 5-HT_2A_ and D_2_ receptors, with the former in regions related to sensorimotor integration (supplementary motor area, superior parietal gyrus, and paracentral lobule) and the latter in most cortical regions. The second cluster identified another distinct profile of correlation coefficients between 5-HT_2A_ receptors in the bilateral hippocampi and D_2_ receptors in most cortical regions.

**Conclusions:**

The observation of two distinct clusters in the correlation matrix suggests regional interactions between 5-HT_2A_ and D_2_ receptors in sensorimotor integration and hippocampal function. A bicluster analysis of the correlation matrix of these neuroreceptors may be beneficial in understanding molecular networks in the human brain.

## Introduction

Serotonin 2A (5-HT_2A_) receptors and dopamine D_2_ receptors are intimately related to the physiology and pathophysiology of neuropsychiatric disorders. Psychotropic agents targeting 5-HT_2A_ and D_2_ receptors are used in the treatment of these disorders [1,2]. In addition to the individual functions of the 5-HT_2A_ and D_2_ receptors, the interactions between 5-HT_2A_ and D_2_ receptors have been studied in the living brain. For example, a 5-HT_2A_ receptor agonist, 2,5-dimethoxy-4-iodoamphetamine, is reported to increase extra-synaptic concentrations of dopamine and noradrenaline in the rat frontal cortex [3]. In addition, a 5-HT_2A_ receptor antagonist modulates dopamine release in rat brain [4]. Moreover, a 5-HT_2A_ receptor inverse agonist, pimavanserin, potentiates haloperidol-induced dopamine release in the medial prefrontal cortex in rats [5]. In humans, a 5-HT_2A_ receptor agonist, psilocybin, induces schizophrenia-like psychosis [6] and alters dopamine release [7,8]. These findings suggest that both dopaminergic and serotonergic neurotransmitter systems jointly contribute to the development of psychiatric symptoms.

Many studies have investigated interactions between the two neurotransmitter systems using pharmacological and genetic techniques. These studies have usually examined the whole brain. However, little is known regarding the regional relationships between the two neurotransmitter systems in individual human brains. Another line of evidence comes from the finding that the genes for 5-HT_2A_ and D_2_ receptors concomitantly modulate physiological prefrontal efficiency during working memory, as assessed using functional magnetic resonance imaging (fMRI) and the response to antipsychotics [9]. However, we do not as yet have any regional information regarding this interaction.

To investigate the regional interactions between the two neurotransmitter systems in the cortex, we investigated the distributions and densities of the two receptor types in the same individuals. As the distributions and densities of the two receptor types are influenced by many factors, such as gene polymorphisms and functional adaptation, they may reflect functional relationships between the two neurotransmitter systems. We hypothesized that the individual variations of 5-HT_2A_ receptor density in cortical regions are correlated with those of D_2_ receptor density in multiple cortical regions, and that regions sharing a specific interaction between the two neurotransmitter systems would share distinct regional correlation profiles. Using positron emission tomography (PET), we investigated regional relationships of density between 5-HT_2A_ and D_2_ receptors and analyzed the profiles of regional correlation in healthy individuals.

## Materials and methods

### Subjects

Seven healthy male volunteers (age: 23 ± 1.5 years, weight: 66 ± 8.1 kg, mean ± standard deviation [SD]) were recruited. The participants were recruited from the registered volunteers in our research institute from December in 2010 to August in 2014. Among twelve volunteers approached, seven participants agreed to participate in the current study. No subject dropped out from the study. All of the subjects were right-handed and had no current or past history of smoking. No subjects reported drug abuse, alcohol abuse, or mental illness. Written informed consent was obtained from all subjects. The Ethics and Radiation Safety Committee of the National Institute of Radiological Sciences approved the study protocol. The study was registered with the University Hospital Medical Information Network Clinical Trials Registry (UMIN000013798).

### MRI procedures

All subjects underwent a 3.0-T MR scan of the brain for anatomical reference. None of the subjects had structural abnormalities. All MRI images were acquired using a MAGNETOM Verio scanner (Siemens AG; Munich, Germany). T1-weighted MR images were acquired using a three-dimensional magnetization-prepared rapid gradient-echo sequence (echo time: 1.9 ms, repetition time: 2,300 ms, flip angle: 9, field of view: 250 mm, acquisition matrix: 256 × 256, slice thickness: 1 mm).

### PET procedures

Each subject underwent two PET scans to visualize serotonin 5-HT_2A_ and dopamine D_2_ receptors. All of the PET scans were performed using an Eminence SET-3000 GCT/X PET scanner (Shimadzu; Kyoto, Japan). For evaluation of density of D_2_ receptors, a 90-minute dynamic PET scan was performed after an injection of [^11^C]FLB 457. The scan protocol consisted of 35 frames and lasted 90 minutes. The injected dose and specific activity were 235 ± 4.8 MBq and 220 ± 68 GBq/μmol at the time of injection, respectively. For evaluation of density of serotonin 5-HT_2A_ receptors, a 90-minute dynamic PET scan was performed after an injection of [^18^F]altanserin. The scan protocol consisted of 33 frames and lasted 90 minutes. The injected dose and specific activity were 191 ± 5.2 MBq and 153 ± 73 GBq/μmol at the time of injection, respectively. Each PET scan was preceded by a transmission scan for attenuation correction using a ^137^Cs source. A head holder was used to minimize head movement. The two scans were performed on separate days (average interval: 20.6 ± 13 months, range 3.4–40.0 months).

For the [^18^F]altanserin PET, arterial blood samples were obtained manually 33 times after the injection of the radioligand to obtain an arterial input function. Each blood sample was centrifuged to obtain plasma and blood cell fractions, and the concentrations of radioactivity in whole blood and plasma were measured. The fractions of the parent compound and its radiometabolites in plasma were determined using high-performance liquid chromatography from 6 samples for each subject.

### Brain image analysis

All PET images were spatially normalized to the standard anatomic orientation. First, all dynamic images were corrected for head motion using PMOD (version 3.6, PMOD Technologies, Zürich, Switzerland). Second, T1-weighted MR images were coregistered to the corresponding PET images. Third, the MR images were spatially normalized and segmented into gray matter, white matter, and cerebrospinal fluid using SPM8 (Wellcome Institute of Neurology, University College of London, UK). Finally, all PET images were spatially normalized to the standard anatomic orientation (the Montreal Neurological Institute 152 standard space; Montreal Neurological Institute; Montreal, QC, Canada) based on the transformation of the MR images.

### Calculation of regional 5-HT_2A_ receptor density

A multi-linear analysis [10] with an arterial input function (concentration of the parent compound in plasma) was used to estimate total distribution volume (*V*_T_). *V*_T_ is equal to the ratio of the concentration of the radioligand in the tissue to that in the plasma at equilibrium. The time point t* after which the model became linear was set to 20 minutes for the multi-linear analysis. Binding potential (*BP*_P_) was then calculated as follows: *BP*_P_ = *V*_T_*—V*_ND_, where *V*_ND_ is the total distribution volume in the cerebellum as reference tissue and 5-HT_2A_ receptor density is negligible, as indicated by autoradiography studies in humans and an *in vivo* displacement study in monkeys [11–13].

### Calculation of regional D_2_ receptor density

The multi-linear reference tissue model method (MRTM2) was used to estimate binding potential (*BP*_ND_) of [^11^C]FLB 457 [14]. The time point t* was set to 1 minute for MRTM2. The parameter *k*_2_′, which is an efflux constant for the reference region, was calculated for each subject using *k*_2_′ from the thalamus, which is a low-noise region, using MRTM. We used the cerebellum as reference tissue because D_2_ receptor density is negligible in this region as indicated by an autoradiography study in human and an *in vivo* displacement study in monkeys [13,15].

### Regions of interest

Regions of interest (ROIs) were defined based on Automated Anatomical Labeling [16]. For each subject, the regions were intersected with individual gray matter images. The intersected ROIs were applied to the parametric PET images to extract binding potentials for each region. We selected seventy-six ROIs to analyze regional *BP* values in the cerebral cortex. The striatum was excluded from the current analysis because D_2_ receptor density in the striatum is too high to quantify using [^11^C]FLB 457. All kinetic analyses were performed using PMOD (version 3.6, PMOD Technologies, Zürich, Switzerland).

### Correlation and clustering analyses

**Fig 1:**
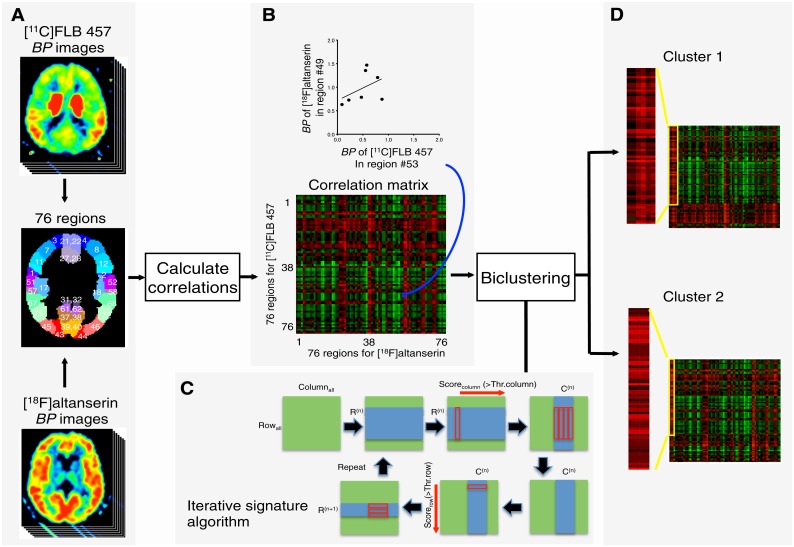
A diagram of procedures of correlation and clustering analyses. A) Individual binding potential (*BP)* values of [^18^F]altanserin and [^11^C]FLB 457 were extracted for 76 regions in seven subjects. B) A correlation matrix of correlation coefficients was calculated between *BP* values of [^18^F]altanserin and [^11^C]FLB 457 for the 76 regions. An example of a scatter diagram (top) shows the correlation between *BP* values of [^11^C]FLB 457 in the left superior parietal gyrus (region #53) and the *BP* values of [^18^F]altanserin in the left fusiform gyrus (region #49). C) Biclustering was performed on the matrix using an iterative signature algorithm (see Methods). R^(n)^ and C^(n)^: sets of rows and columns at the n^th^ iteration, Score_row_ and Score_column_: the evaluation scores for the rows and columns, Thr.row and Thr.column: thresholds for the rows and columns. D) Two clusters of regions were identified (see Results).

Procedures of correlation and clustering analyses were summarized in a diagram (Fig 1A–1D). Based on data from the seven subjects, we calculated a correlation matrix of Spearman’s rank correlation coefficients between the *BP*_P_ of [^18^F]altanserin and *BP*_ND_ of [^11^C]FLB 457 for the 76 regions (the two parameters are referred to as *BP* hereafter). The relationships between the distributions of the 5-HT_2A_ and D_2_ receptors were investigated using unsupervised biclustering of regions on the correlation matrix with an iterative signature algorithm (ISA) [17] implemented in the biclustering analysis toolbox (Eidgenössische Technische Hochschule, Zürich). This biclustering method (simultaneous two-directional clustering) identifies regions of distinct regional profiles of correlation coefficients for 5-HT_2A_ and D_2_ receptors by optimizing thresholds described previously [17,18]. In brief, we determined biclusters that were initially selected from 50 random starting points across the rows and columns of the correlation matrix and were iteratively refined to satisfy a given set of thresholds using the following steps (Fig 1C). Step 1: The ISA initially started with a set of random regions (R^(n)^, n = 0) selected from all of the rows. Step 2: An evaluation score for the columns (Score_column_) was calculated as an average of correlation coefficients in the extracted column multiplied by the score for the row (Score_row_). Step 3: A set of columns (C^(n)^, n = 0) was extracted from all columns so that the Score_column_ was larger than a preset threshold for the column. Step 4: Score_row_ was re-calculated as an average of the correlation coefficients in the extracted rows multiplied by Score_column_. Step 5: A set of rows (R^(n)^, n = 1) was extracted from all rows so that Score_row_ was larger than a preset threshold for the row. Finally, steps 2 to 5 were repeated until convergence (R^(n)^ = R^(n+1)^) was achieved. R^(n)^ and C^(n)^ were then reported as clusters. We then optimized the thresholds for the identification of the biclusters [18]. The optimization of the thresholds attempted to rigorously extract biclusters with as high a number of regions as possible without overlapping regional pairs among the biclusters. The optimized thresholds for rows and columns were 1.4 and 2.0, respectively.

## Results

**Fig 2:**
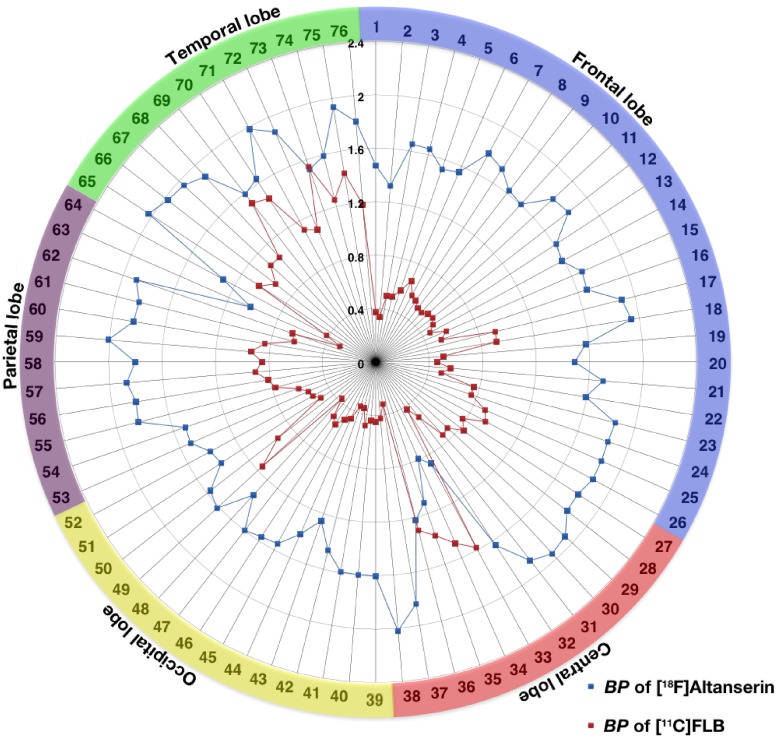
Regional binding potential (*BP*) values for 5-HT_2A_ and D_2_ receptors in cerebral cortical regions. Mean *BP* values for [^18^F]altanserin for 5-HT_2A_ receptors (blue) and [^11^C]FLB 457 for D_2_ receptors (red) were plotted for 76 cerebral regions. The numbers represent regions defined in Table 1. Raw data of *BP* values is available as supporting information (S1 File).

5-HT_2A_ receptors were distributed in widespread regions of cerebral cortex, while D_2_ receptors had varying densities in the 76 cerebral cortical regions (Fig 2). The mean regional *BP* values of 5-HT_2A_ receptors were between 0.8 and 2.0, being relatively low in the hippocampus (0.83 ± 0.34), paracentral lobule (1.16 ± 0.33), and parahippocampus (1.17 ± 0.35) (Fig 2). In contrast, the mean *BP* values of D_2_ receptors, varied among the different regions. The *BP* values were high in the hippocampus (1.53 ± 0.25), parahippocampus (1.34 ± 0.24), and temporal cortex (1.23 ± 0.36) and low in the paracentral lobule (0.36 ± 0.19), occipital lobe (0.43 ± 0.20), and frontal cortex (0.51 ± 0.21) (Fig 2). The observed regional distributions of 5-HT_2A_ and D_2_ receptors were consistent with the findings of previous postmortem [19] and PET studies [20–22].

**Table 1:** Brain regions used for the correlation matrix analysis.

Frontal lobe	Central regions	Parietal lobe
1, 2. precentral gyrus	27,28. anterior cingulum	53,54. superior parietal gyrus
3,4. superior frontal gyrus	29,30. middle cingulum	55,56. inferior parietal gyrus
5,6. superior frontal gyrus, orbital	31,32. posterior cingulum	57,58. supramarginal gyrus
7,8. middle frontal gyrus	33,34. hippocampus	59,60. angular gyrus
9,10. middle frontal gyrus, orbital	35,36. parahippocampus	61,62. precuneus
11,12. inferior frontal gyrus, opercular	37,38. calcarine fissure and surrounding cortex	63,64. paracentral lobule
13,14. inferior frontal gyrus, triangular	Occipital lobe	Temporal lobe
15,16. inferior frontal gyrus, orbital	39,40. cuneus	65,66. Heschl gyrus
17,18. Rolandic operculum	41,42. lingual gyrus	67,68. superior temporal gyrus
19,20. supplementary motor area	43,44. superior occipital lobe	69,70. temporal pole, superior temporal gyrus
21,22. superior frontal gyrus, medial	45,46. middle occipital lobe	71,72. middle temporal gyrus
23,24. medial frontal gyrus, orbital	47,48. inferior occipital lobe	73,74. temporal pole, middle temporal gyrus
25,26. gyrus rectus	49,50. fusiform gyrus	75,76. inferior temporal gyrus
	51,52. postcentral gyrus	

Odd and even numbers correspond to left and right regions, respectively.

**Fig 3:**
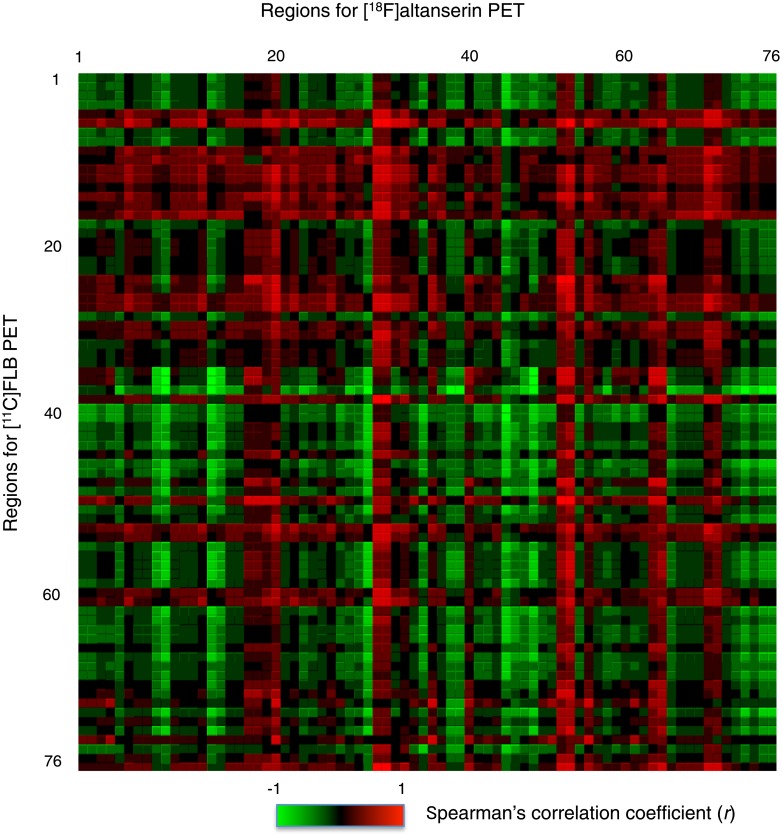
Correlation matrix of regions containing 5-HT_2A_ and D_2_ receptors. A correlation matrix was generated based on Spearman’s correlation coefficients (*r* values) between individual binding potential values of [^18^F]altanserin for 5-HT_2A_ receptors (columns) and [^11^C]FLB457 for D_2_ receptors (rows) in 76 regions. The numbers represent regions defined in Table 1. Raw data of the matrix is available as supporting information (S2 File).

**Fig 4:**
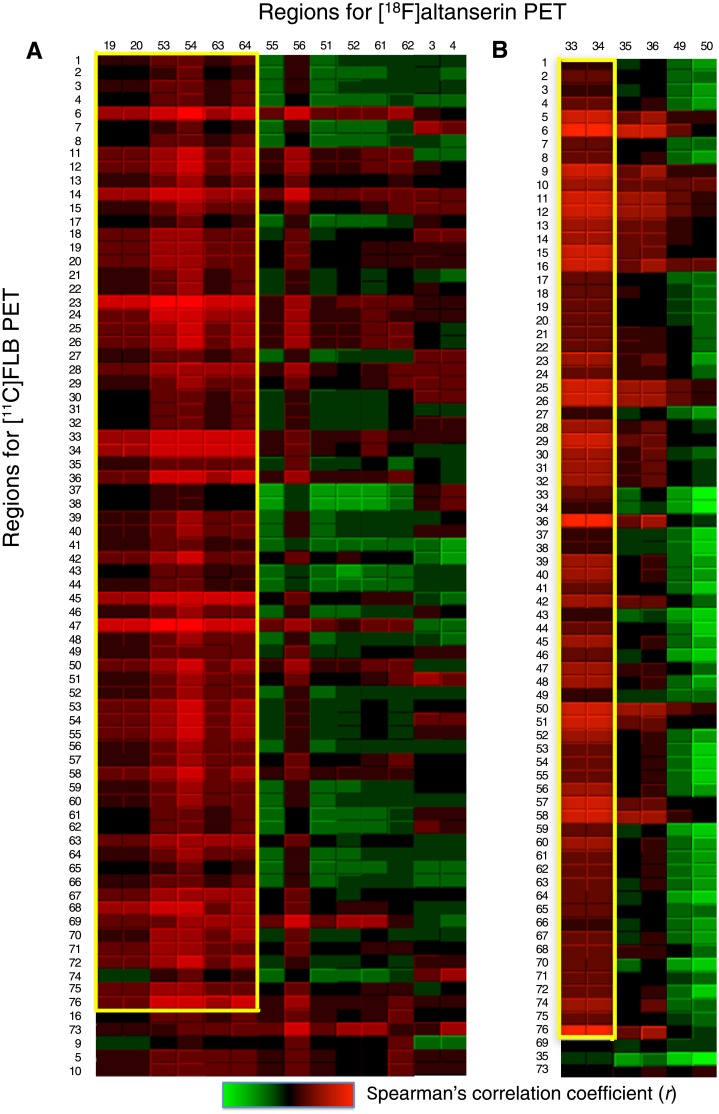
Correlation matrix of regions extracted as clusters using the biclustering analysis and their adjacent regions (outside of the yellow boxes). (A) The first cluster consisted of correlations between 5-HT_2A_ receptor binding potentials (*BP*) for the frontal and parietal cortices and D_2_ receptor *BP* for broad cortical regions (71 regions). Regions in the first cluster (inside the yellow boxes): supplementary motor area (19, 20), superior parietal gyrus (53, 54), paracentral lobule (63, 64); regions adjacent to the first cluster (outside of the yellow boxes): inferior parietal gyrus (55, 56), postcentral gyrus (51, 52), precuneus (61, 62), and superior frontal gyrus (3, 4). (B) The second cluster consisted of correlations between 5-HT_2A_ receptor *BP* for the bilateral hippocampi and D_2_ receptor *BP* for broad regions (73 regions) in the cerebral cortex. Regions in the second cluster (inside the yellow boxes): hippocampus (33, 34), regions adjacent to the second cluster (outside of the yellow boxes): parahippocampus (35, 36) and fusiform (49, 50).

**Fig 5:**
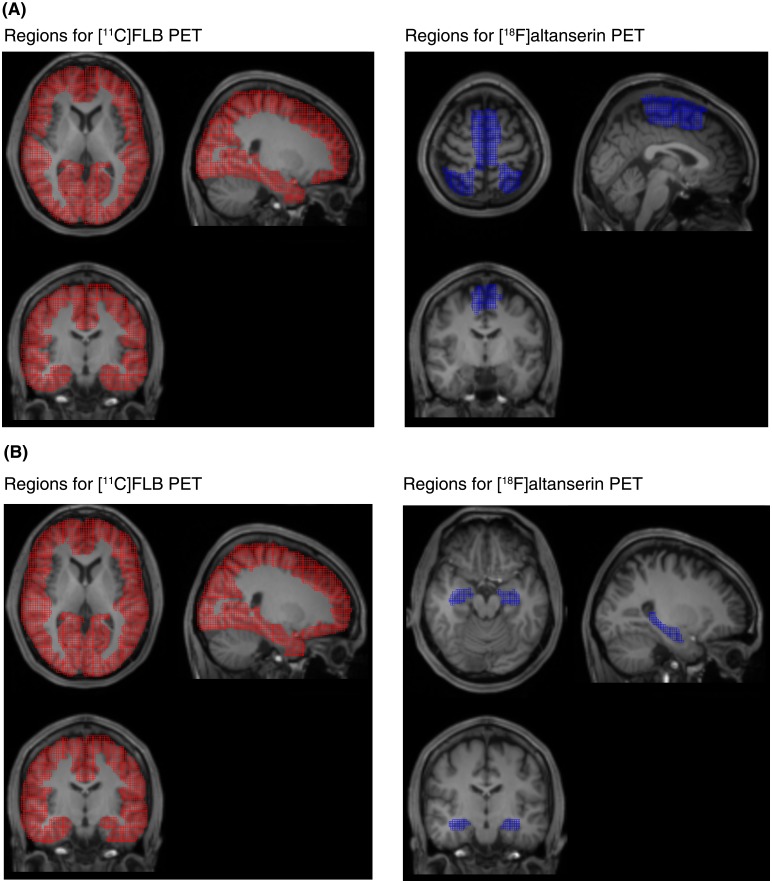
Each atlas shows regional pairs extracted as clusters using the bicluster analysis. **(A)** Regions for 5-HT_2A_ receptors and those for D_2_ receptors in the first cluster. **(B)** Regions for 5-HT_2A_ receptors and those for D_2_ receptors in the second cluster.

The bicluster analysis of the correlation matrix of the 76 regions (Fig 3), identified two clusters of regions (Figs 4 and 5), which were characterized by distinctive profiles of regional 5-HT_2A_ and D_2_ receptor density correlations pairs. The two clusters were mutually exclusive, and they resulted to consist of positive correlation coefficients.

The first cluster consisted of regional pairs with positive correlation coefficients between 5-HT_2A_ receptors in the supplementary motor area, superior parietal gyrus, and paracentral lobule and D_2_ receptors in broad cortical regions (*r* = 0.40 ± 0.17 [mean ± SD], Figs 4A and 5A). 5-HT_2A_ receptors in the adjacent regions (inferior parietal gyrus, postcentral gyrus, precuneus, and superior frontal gyrus), which were not extracted in the first cluster, had quite different profiles of correlation coefficients against D_2_ receptors in the broad cortical regions. These regions included regional pairs with negative correlation coefficients, as indicated by the green boxes. D_2_ receptors in the five regions (orbital part of the bilateral middle frontal gyri, left superior frontal gyrus, right inferior frontal gyrus, and left temporal pole in the middle temporal gyrus) were not extracted into the first cluster.

The second cluster consisted of regional pairs with positive correlation coefficients between 5-HT_2A_ receptors in the bilateral hippocampi and D_2_ receptors in broad cortical regions (*r* = 0.48 ± 0.15, mean ± SD, Figs 4B and 5B). 5-HT_2A_ receptors in the adjacent regions (parahippocampus and fusiform), which were not extracted in the second cluster, had negative correlation coefficients against D_2_ receptors in the broad cortical regions. D_2_ receptors in the temporal pole in the superior and middle temporal gyri and the parahippocampus were not extracted into the second cluster.

## Discussion

In this study, we identified two clusters of regions with distinct regional profiles of correlation coefficients between 5-HT_2A_ receptors and D_2_ receptors in the matrix. The first cluster suggests that 5-HT_2A_ receptors in the regions of sensorimotor integration (supplementary motor area, superior parietal gyrus, and paracentral lobule) may have interactions with D_2_ receptors in the broad cortical regions. The second cluster indicates that 5-HT_2A_ receptors in the bilateral hippocampi may have other interactions with D_2_ receptors in the broad cortical regions.

Individual variations in the densities of the receptors may be caused by their baseline expressions and adaptation [23]. Under the situation where gene expression and adaptation interact and/or are regulated simultaneously in two separated regions, the densities of receptors may correlate between the two regions. This would suggest functional associations between the two receptor types in the two regions. In the human cortex, 5-HT_2A_ receptors are mainly expressed post-synaptically on glutamatergic pyramidal neurons, cholinergic neurons, and gamma-aminobutyric acid (GABA)ergic interneurons [24,25]. In contrast, D_2_ receptors are mainly expressed on presynaptic dopaminergic neurons and postsynaptic GABAergic interneurons [26]. The functional associations between the two receptors in the separate regions may reflect the direct and/or indirect projections of those neurons from the origins of the serotonin and dopamine systems, which are the raphe nucleus and the ventral tegmental area, respectively. In this study, we performed a bicluster analysis of the correlation matrix of individual variation in the two receptor densities. We identified distinct regional correlation profiles between the two receptor types and extracted clusters that reflected functional interrelationships between the two neurotransmitter systems in the extracted regions.

In the first cluster, the extracted regions containing 5-HT_2A_ receptors were related to sensorimotor integration. The supplementary motor area is involved in the integration of motor functions [27,28] and the superior parietal gyrus is involved in the integration of somatosensory and visual functions [29]. The paracentral lobule integrates motor and sensory functions with motor and sensory innervation of the contralateral lower extremity [30]. 5-HT_2A_ receptors are involved in the above sensorimotor processes, which may be disrupted in schizophrenia and affected by hallucinogens [31,32]. As a modification of the dopamine system by D_2_ receptors can also affect sensorimotor processing [33], the current cluster may indicate regions with interactions related to sensorimotor integration between the two neurotransmitter systems.

In the second cluster, the extracted regions containing 5-HT_2A_ receptors were the bilateral hippocampi. The hippocampus is a key region in the brain for memory processing. 5-HT_2A_ receptor activation in the hippocampus facilitates the consolidation of object memory [34, 35], fear learning [36], and long-term memory [37]. As the function of the mesocortical dopamine pathway is not limited to executive functions such as working memory, but also involves aversion and learning [38], our results may reflect the interactions between the two neurotransmitter systems in memory functions.

The results of clustering analysis, which was carried out using an iterative signature algorithm, depend on the optimization of thresholds. In this study, we optimized thresholds based on a standard method reported previously [18]. We used the numbers of extracted columns, extracted rows and identified clusters as parameters of the optimization. These parameters were plotted graphically for various threshold values of rows and columns. Threshold values leading to the retention of as much coverage and rigidity as possible were chosen in a fixed range based on graphic judgments. The numbers of the extracted clusters did not change when the thresholds were altered within their ranges. This reflects the robustness of the results to variation in the thresholds. However, a small number of regions containing D_2_ receptors in the extracted clusters varied in the optimization process used for the thresholds. In clusters 1 and 2, most of the cortical regions were included in the clusters at the D_2_ receptor side of regional pairs, while 3 to 5 regions were excluded from the clusters. As the numbers of regions excluded from the clusters were not stable when we changed the thresholds in the narrowed range, we did not consider the regions excluded from the clusters at the D_2_ receptor side of regional pairs as significant.

The small sample size is a limitation of the current study, and any generalization of our findings needs to be approached with caution. An additional study with a larger sample size is needed to replicate and confirm the current findings. Another limitation is that our study included only male subjects. Any potential effect of gender on our current findings needs to be addressed in future studies. Finally, the large time difference between two PET scans is also a limitation of the current study. However, we believe the effects of the PET scan time difference on the results of clustering should be minimum if any for the following reasons. Previous PET studies have shown that *BP* of [^11^C]FLB 457 changes slowly with aging (10% decrease per decade) [39], and *BP* of [^18^F]altanserin also changes slowly with aging (less than 10% decrease per decade) [40]. The PET scan time differences in our study were between 3 and 40 months, which would correspond to potential *BP* changes if any due to the scan time differences are within 5%, being less than the test-retest variability of the PET measurements of usually 10% or more.

Our approach may be useful not only for the analysis of the interaction between different brain system, but also for understanding dysfunction in neurotransmitter systems. For example, in patients with the obsessive compulsive disorder, a significant reduction in 5-HT_2A_ receptor density is observed in the cortex, including the associative area [13]. In addition, antipsychotic augmentation is effective in some patients refractory to serotonin reuptake inhibitors [41]. In patients with Alzheimer’s disease, 5-HT_2A_ receptor density was reduced in most neocortical areas [42]. In rodent models, the Alzheimer’s-like neuropathology and memory were associated with a reduction of hippocampal 5-HT_2A_ receptor expression [43]. In aging, 5-HT_2A_ receptor density slowly decreases [40]. Since those studies did not investigate the density of dopamine D_2_ receptors in the same subjects, it is not possible to assess the alteration of the interaction between the two neurotransmitter systems in such diseases or aging. However, our study will provide an opportunity to consider simultaneous observation of the two neurotransmitter systems to see their potential interactions in the regions in clusters in future studies. It would be helpful to know whether interactions between the two neurotransmitter systems in the extracted cluster regions contribute to such disorders.

## Conclusions

In conclusion, we identified two regional clusters that may suggest regional interactions between 5-HT_2A_ and D_2_ receptors in sensorimotor integration and hippocampal functions. Bicluster analysis of the correlation matrix of neuroreceptors may be beneficial in understanding molecular networks in the human brain.

## Supporting information

S1 File Regional binding potential (*BP*) values for 5-HT_2A_ and D_2_ receptors in cerebral cortical regions. An excel file contains *BP* vales of [^18^F]altanserin for 5-HT_2A_ receptors and [^11^C]FLB 457 for D_2_ receptors in cerebral cortical regions.
(XLSX)

S2 File Correlation matrix of regions containing 5-HT_2A_ and D_2_ receptors. An excel file contains Spearman’s correlation coefficients (*r* values) between individual binding potential values of [^18^F]altanserin for 5-HT_2A_ receptors (columns) and [^11^C]FLB 457 for D_2_ receptors (rows) in 76 regions.
(XLSX)
