# Correction: Anatomical relationships between serotonin 5-HT_2A_ and dopamine D_2_ receptors in living human brain

**DOI:** 10.1371/journal.pone.0197201

**Published:** 2018-05-07

**Authors:** Tatsuya Ishii, Yasuyuki Kimura, Masanori Ichise, Keisuke Takahata, Soichiro Kitamura, Sho Moriguchi, Manabu Kubota, Ming-Rong Zhang, Makiko Yamada, Makoto Higuchi, Yoshiro Okubo, Tetsuya Suhara

The eleventh author’s name is spelled incorrectly. The correct name is: Yoshiro Okubo.
